# The future of sensory substitution, addition, and expansion via haptic devices

**DOI:** 10.3389/fnhum.2022.1055546

**Published:** 2023-01-13

**Authors:** David M. Eagleman, Michael V. Perrotta

**Affiliations:** ^1^Department of Psychiatry, Stanford University School of Medicine, Stanford, CA, United States; ^2^Neosensory, Palo Alto, CA, United States

**Keywords:** senses, sensory substitution devices, hearing loss, haptics, sound

## Abstract

Haptic devices use the sense of touch to transmit information to the nervous system. As an example, a sound-to-touch device processes auditory information and sends it to the brain via patterns of vibration on the skin for people who have lost hearing. We here summarize the current directions of such research and draw upon examples in industry and academia. Such devices can be used for sensory substitution (replacing a lost sense, such as hearing or vision), sensory expansion (widening an existing sensory experience, such as detecting electromagnetic radiation outside the visible light spectrum), and sensory addition (providing a novel sense, such as magnetoreception). We review the relevant literature, the current status, and possible directions for the future of sensory manipulation using non-invasive haptic devices.

## Introduction

The endeavor of getting information to the brain via unusual channels has a long history. We here concentrate on non-invasive devices that use haptics, or the sense of touch. In recent years, as computing technology has advanced, many haptic-based devices have been developed. We categorize these devices into three groups based on their function: sensory substitution, sensory expansion, and sensory addition.

The key to understanding the success of haptics requires remembering that the brain does not directly hear or see the world. Instead, the neural language is built of electrochemical signals in neurons which build some representation of the outside world. The brain's neural networks take in signals from sensory inputs and extract informationally-relevant patterns. It strives to adjust to whatever information it receives and works to extract what it can. As long as the data reflects some relevant feature about the outside world, the brain works to decode it (Eagleman, 2020). In this sense, the brain can be viewed as a general-purpose computing device: it absorbs the available signals and works to determine how to optimally make use of them.

## Sensory substitution

Decades ago, researchers realized that the brain's ability to interpret different kinds of incoming information implied that one might be able to get one sensory channel to carry another's information (Bach-y-Rita et al., 1969). In a surprising demonstration, Bach-y-Rita et al. placed blind volunteers in a reconfigured dental chair in which a grid of four hundred Teflon tips could be extended and retracted by mechanical solenoids. Over the blind participant a camera was mounted on a tripod. The video stream of the camera was converted into a poking of the tips against the volunteer's back. Objects were passed in front of the camera while blind participants in the chair paid careful attention to the feelings in their backs. Over days of training, they became better at identifying the objects by their feel. The blind subjects learned to distinguish horizontal from vertical from diagonal lines, and more advanced users could learn to distinguish simple objects and even faces—simply by the tactile sensations on their back. Bach-y-Rita's findings suggested that information from the skin can be interpreted as readily (if with lower resolution) as information coming from the eyes, and this demonstration opened the floodgates of sensory substitution (Hatwell et al., 2003; Poirier et al., 2007; Bubic et al., 2010; Novich and Eagleman, 2015; Macpherson, 2018).

The technique improved when Bach-y-Rita and his collaborators allowed the blind user to point the camera, using his own volition to control where the “eye” looked (Bach-y-Rita, 1972, 2004). This verified the hypothesis that sensory input is best learned when one can interact with the world. Letting users control the camera closed the loop between muscle output and sensory input (Hurley and Noë, 2003; Noe, 2004). Perception emerges not from a passive input, but instead as a result of actively exploring the environment and matching particular actions to specific changes in sensory inputs. Whether by moving extraocular muscles (as in the case of sighted people) or arm muscles (Bach-y-Rita's participants), the neural architecture of the brain strives to figure out how the output maps to subsequent input (Eagleman, 2020).

The subjective experience for the users was that objects captured by the camera were felt to be located at a distance instead of on the skin of the back (Bach-y-Rita et al., 2003; Nagel et al., 2005). In other words, it was something like vision: instead of stimulating the photoreceptors, the information stimulated touch receptors on the skin, resulting in a functionally similar experience.

Although Bach-y-Rita's vision-to-touch system was the first to seize the public imagination, it was not the first attempt at sensory substitution. In the early 1960s, Polish researchers had passed visual information via touch, building a system of vibratory motors mounted on a helmet that “drew” the images on the head through vibrations [the *Elektroftalm*; (Starkiewicz and Kuliszewski, 1963)]. Blind participants were able to navigate specially prepared rooms that were painted to enhance the contrast of door frames and furniture edges. Unfortunately, the device was heavy and would get hot during use, and thus was not market-ready—but the proof of principle was there.

These unexpected approaches worked because inputs to the brain (such as photons at the eyes, air compression waves at the ears, pressure on the skin) are all converted into electrical signals. As long as the incoming spikes carry information that represents something important about the outside world, the brain will attempt to interpret it.

In the 1990s, Bach-y-Rita et al. sought ways to go smaller than the dental chair. They developed a small device called the BrainPort (Bach-y-Rita et al., 2005; Nau et al., 2015; Stronks et al., 2016). A camera is attached to the forehead of a blind person, and a small grid of electrodes is placed on the tongue. The “Tongue Display Unit” of the BrainPort uses a grid of stimulators over three square centimeters. The electrodes deliver small shocks that correlate with the position of pixels, feeling something like Pop Rocks candy in the mouth. Bright pixels are encoded by strong stimulation at the corresponding points on the tongue, gray by medium stimulation, and darkness by no stimulation. The BrainPort gives the capacity to distinguish visual items with a visual acuity that equates to about 20/800 vision (Sampaio et al., 2001). While users report that they first perceive the tongue stimulation as unidentifiable edges and shapes, they eventually learn to recognize the stimulation at a deeper level, allowing them to discern qualities such as distance, shape, direction of movement, and size (Stronks et al., 2016).

The tongue provides an excellent brain-machine interface because it is densely packed with touch receptors (Bach-y-Rita et al., 1969; Bach-y-Rita, 2004). When brain imaging is performed on trained subjects (blind or sighted), the motion of electrotactile shocks across the tongue activates the MT+ area of the visual cortex, an area which is normally involved in visual motion (Merabet et al., 2009; Amedi et al., 2010; Matteau et al., 2010).

Of particular interest is the subjective experience. The blind participant Roger Behm describes the experience of the BrainPort:

Last year, when I was up here for the first time, we were doing stuff on the table, in the kitchen. And I got kind of... a little emotional, because it's 33 years since I've seen before. And I could reach out and see the different-sized balls. I mean I visually see them. I could reach out and grab them—not grope or feel for them—pick them up, and see the cup, and raise my hand and drop it right in the cup (Bains, 2007).

Tactile input can work on many locations on the body. For example, the Forehead Retina System converts a video stream into a small grid of touch on the forehead (Kajimoto et al., 2006). Another device hosts a grid of vibrotactile actuators on the abdomen, which use intensity to represent distance to the nearest surfaces. Researchers used this device to demonstrate that blind participants' walking trajectories are not preplanned, but instead emerge dynamically as the tactile information streams in Lobo et al. (2017, 2018).

As 5% of the world has disabling hearing loss, researchers have recently sought to build sensory substitution for the deaf (Novich and Eagleman, 2015). Assimilating advances in high-performance computing into a sound-to-touch sensory-substitution device worn under the shirt, Novich and Eagleman (2015) built a vest that captured sound around the user and mapped it onto vibratory motors on the skin, allowing users to feel the sonic world around them. The theory was to transfer the function of the inner ear (breaking sounds into different frequencies and sending the data to the brain) to the skin.

**Figure 1:**
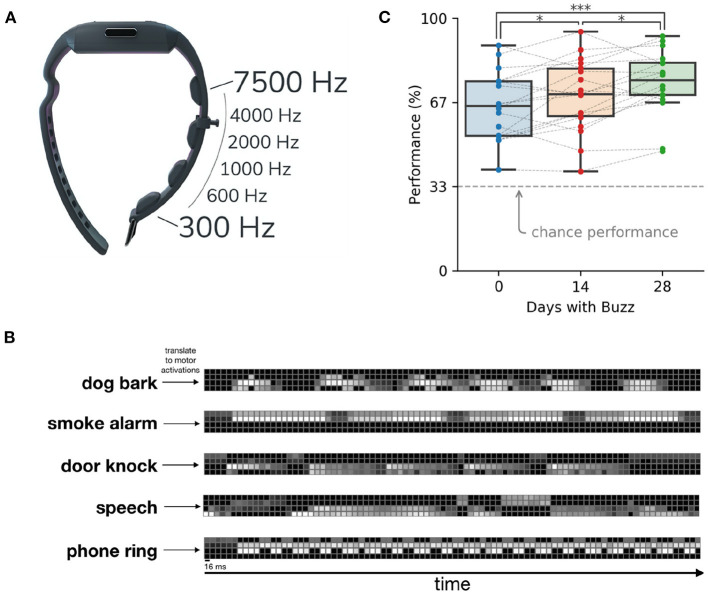
A sensory substitution wristband for deafness (the Neosensory Buzz). **(A)** Four vibratory motors in a wristband transfer information about sound levels in different frequency bands. **(B)** Sample sounds translated into patterns of vibration on the motors of the wristband. Brighter colors represent higher intensity of the motor. **(C)** Participant performance on sound identification improves through time. Data reprinted from Perrotta et al. (2021).

Does the skin have enough bandwidth to transmit all the information of sound? After all, the cochlea is an exquisitely specialized structure for capturing sound frequencies with high fidelity, while the skin is focused on other measures and has poor spatial resolution. Conveying a cochlear-level of information through the skin would require several thousand vibrotactile motors—too many to fit on a person. However, by compressing the speech information, just a few motors suffice (Koffler et al., 2015; Novich and Eagleman, 2015). Such technology can be designed in many different form factors, such as a chest strap for children and a wristband with vibratory motors (Figure 1).

On the first day of wearing the wristband, users are at the very least able to use the vibrations as cues that a noise is happening. Users quickly learn to use the vibrations to differentiate sounds, such as a dog barking, a faucet running, a doorbell ringing, or someone calling their name. A few days into wearing the wristband, users report that the conscious perception of the vibrations fades into the background but still aids them in knowing what sounds are nearby. Users' ability to differentiate patterns of vibrations improves over time. Figure 1C shows performance scores over time on a three-alternative forced choice paradigm, where the three choices were picked at random from a list of 14 environmental sounds. One environmental sound was presented as vibrations on the wristband and the user had to choose which sound they felt (Perrotta et al., 2021).

Moreover, after several months users develop what appears to be a direct subjective experience of external sound. After 6 months, one user reported that he no longer has a sensation of buzzing followed by an interpretation of those vibrations, but instead, “I perceive the sound in my head” (personal interview). This was a subjective report and qualia are not possible to verify; nonetheless we found the claim sufficiently interesting to note here.

The idea of converting touch into sound is not new (Traunmüller, 1980; Cholewiak and Sherrick, 1986; Weisenberger et al., 1991; Summers and Gratton, 1995; Galvin et al., 2001; Reed and Delhorne, 2005). In 1923, Robert Gault, a psychologist at Northwestern University, heard about a deaf and blind ten-year-old girl who claimed to be able to feel sound through her fingertips. Skeptical, he ran experiments. He stopped up her ears and wrapped her head in a woolen blanket (and verified on his graduate student that this prevented the ability to hear). She put her finger against the diaphragm of a “portophone” (a long hollow tube), and Gault sat in a closet and spoke through it. Her only ability to understand what he was saying was from vibrations on her fingertip. He reports,

After each sentence or question was completed her blanket was raised and she repeated to the assistant what had been said with but a few unimportant variations.... I believe we have here a satisfactory demonstration that she interprets the human voice through vibrations against her fingers.

Gault mentions that his colleague has succeeded at communicating words through a thirteen-foot-long glass tube. A trained participant, with stopped-up ears, could put his palm against the end of the tube and identify words that were spoken into the other end. With these sorts of observations, researchers have attempted to make sound-to-touch devices, but until recent decades the machinery was too large and computationally weak to make for a practical device.

Similarly, the Tadoma method, developed in the 1930s, allows people who are deaf and blind to understand the speech of another person by placing a hand over the face and neck of the speaker. The thumb rests lightly on the lips and the fingers fan out to cover the neck and cheek, allowing detection of moving lips, vibrating vocal cords, and air coming out of the nostrils. Thousands of deaf and blind children have been taught this method and have obtained proficiency at understanding language almost to the point of those with hearing, all through touch (Alcorn, 1945).

In the 1970s, deaf inventor Dimitri Kanevsky developed a two-channel vibrotactile device, one of which captures the envelope of low frequencies, and the other high. Two vibratory motors sit on the wrists. By the 1980s, similar inventions in Sweden and the United States were proliferating. The problem was that all these devices were too large, with too few motors (typically just one) to make an impact. Due to computational limitations in previous years, earlier attempts at sound-to-touch substitution relied on band-pass filtering audio and playing this output to the skin over vibrating solenoids. The solenoids operated at a fixed frequency of less than half the bandwidth of some of these band-passed channels, leading to aliasing noise. Further, multichannel versions of these devices were limited in the number of actuators due to battery size and capacity constraints. With modern computation, the desired mathematical transforms can be performed in real time, at little expense, and without the need of custom integrated circuits, and the whole device can be made as an inexpensive, wearable computing platform.

A wrist-worn sound-to-touch sensory substitution device was recently shown in brain imaging to induce activity in both somatosensory and auditory regions, demonstrating that the brain rapidly recruits existing auditory processing areas to aid in the understanding of the touch (Malone et al., 2021).

There are cost advantages to a sensory substitution approach. Cochlear implants typically cost around $100,000 for implantation (Turchetti et al., 2011). In contrast, haptic technologies can address hearing loss for some hundreds of dollars. Implants also require an invasive surgery, while a vibrating wristband is merely strapped on like a watch.

There are many reasons to take advantage of the system of touch. For example, people with prosthetic legs have difficulty learning how to walk with their new prosthetics because of a lack of proprioception. To allow participants to understand the position of an artificial limb, other sensory devices can channel the information. For example, research has shown improvements in stair descent for lower limb amputees using haptic sensory substitution devices (Sie et al., 2017), and haptic sensory substitution devices have also been created for providing sensory feedback for upper limb prosthetics (Cipriani et al., 2012; Antfolk et al., 2013; Rombokas et al., 2013). Some sensory substitution devices for upper limb amputees use electrotactile stimulation instead of vibrotactile stimulation, targeting different receptors in the skin (Saleh et al., 2018).

This same technique can be used for a person with a real leg that has lost sensation—as happens in Parkinson's disease or peripheral neuropathy. In unpublished internal experiments, we have successfully piloted a solution that used sensors in a sock to measure motion and pressure and fed the data into the vibrating wristband. By this technique, a person understands where her foot is, whether her weight is on it, and whether the surface she's standing on is even. A recent systematic review synthesizing the findings of nine randomized controlled trials showed that sensory substitution devices are effective in improving balance measures of neurological patient populations (Lynch and Monaghan, 2021).

Touch can also be used to address problems with balance. This has been done with the BrainPort tongue display (Tyler et al., 2003; Danilov et al., 2007): the head orientation was fed to the BrainPort tongue grid: when the head was straight up, the electrical stimulation was felt in the middle of the tongue grid; when the head tilted forward, the electrical signal moved toward the tip of the tongue; when the head tilted back, the stimulation moved toward the rear; side-to-side tilts were encoded by left and right movement of the electrical signal. In this way, a person who had lost all sense of which way her head was oriented could feel the answer on her tongue. Of note, the residual benefits extended even after taking off the device. Users' brains figured out how to take residual vestibular signals as well as existing visual and proprioceptive signals and strengthen them with the guidance of the helmet. After several months of using the helmet, many participants were able to reduce the frequency of their usage.

We have developed a similar experimental system that is currently unpublished but bears mentioning for illustration purposes. In a balance study underway at Stanford University, we use a vibratory wristband in combination with a 9-axis inertial measurement unit (IMU) that is clipped to the collar of a user. The IMU outputs an absolute rotation relative to a given origin, which is set as the device's position when the user is standing upright. The pitch and roll of the rotation are mapped to vibrations on the wristband to provide additional balance information to the user's brain: the more one tilts away from upright, the higher the amplitude of vibrations one feels on the wrist. The direction of the tilt (positive or negative pitch or roll) is mapped to the vibration location on the wrist. Results of this approach will be published in the future.

Besides the basic five senses, more complex senses can be aided with sensory substitution devices. People with autism spectrum disorder often have a decreased ability to detect emotion in others; this, in one preliminary project, machine learning algorithms classify the emotional states detected in speech and communicate these emotional states to the brain via vibrations on the wrist. Currently, the machine learning algorithm detects and communicates how much someone's speech matches seven different emotions (neutral, surprise, disgust, happiness, sadness, fear, and anger) and communicates that to the wearer (ValenceVibrations.com).

Sensory substitution opens new opportunities to compensate for sensory loss. However, similar devices can move past compensation and instead build on top of normal senses—we call these devices sensory expansion devices. Instead of filling in gaps for someone with a sensory deficit, these expand the unhindered senses to be better, wider, or faster.

## Sensory expansion

Many examples of sensory expansion have been demonstrated in animals. For example, mice and monkeys can be moved from color-blindness to color vision by genetically engineering photoreceptors to contain human photopigment (Jacobs et al., 2007; Mancuso et al., 2009). The research team injected a virus containing the red-detecting opsin gene behind the retina. After 20 weeks of practice, the monkeys could use the color vision to discriminate previously indistinguishable colors.

In a less invasive example, we created a sensory expansion device by connecting a vibrating wristband to a near-wavelength infrared sensor and an ultraviolet sensor. Although our eyes capture only visible light, the frequencies of the electromagnetic spectrum adjacent to visible light are in fact visible to a variety of animals. For instance, honeybees can see ultraviolet patterns on flowers (Silberglied, 1979). By capturing the intensity of light in these ranges and mapping those intensities to vibrations, a user can pick up on information in these invisible light regions without gene editing or retinal implants. In this way, a wrist-worn device can expand vision beyond its natural capabilities. One of us (DME) wore an infrared bolometer connected to a haptic wristband and was able to easily detect infrared cameras in the darkness (Eagleman, 2020).

To illustrate the breadth of possibilities, it bears mention that we have performed an unpublished preliminary experiment with blindness. Using lidar (light detection and ranging), we tracked the position of every moving object in an office space—in this case, humans moving around. We connected the data from the lidar sensors to our vibrating vest, such that the vest vibrated to tell the wearer if they were approaching an obstacle like a wall or chair, where there were people nearby, and what direction they should move to most quickly reach a target destination. We tested this sensory expansion device with a blind participant. He wore the vest and could feel the location of objects and people around him as well as the quickest path to a desired destination (such as a conference room). Interestingly, there was no learning curve: he immediately understood how to use the vibrations to navigate without colliding into objects or people. Although sensory substitution devices can fill the gap left by vision impairment, this device did more than that—it offered an expanded, 360° sense of space. A sighted person could also wear this device to expand their sense of space, allowing them to know what objects or people are behind them. Because this device does more than alleviate a sensory loss, it is an example of sensory expansion.

Haptic sensory expansion is not limited to vision. Devices from hearing aids to the Buzz can reach beyond the normal hearing scale—for example, into the ultrasonic range (as heard by cats or bats), or the infrasonic (as heard by elephants) (Wolbring, 2013).

The sense of smell can also be benefited by sensory expansion. To illustrate an unpublished possibility, imagine converting the data from an array of molecular detectors into haptic signals. While this is unproven, the goal should be clear: for a person to access a new depth of odor detection, beyond the natural sensory acuity of human smell.

One can also detect temperature via sensory expansion. In preliminary experiments, participants use an array of mid-wavelength infrared sensors to detect the temperature of nearby objects and translate the data to vibrations on the wrist. The wearer learns to interpret the vibrations as a sense of temperature, but one that does not stop at the skin—instead, their sense of temperature has expanded to include objects in the surrounding environment.

For the purpose of illustrating the width of possibilities, we note that internal signals in the body—such as the sense of one's own blood sugar levels—can be easily expanded by combining easily-obtainable technologies. For example, continuous glucose monitoring devices allow users to look at their level of blood sugar at any point; however, the user still must pull out a cell phone to consult an app. By connecting a haptic device to a continuous glucose monitoring device, one could create a sensory expansion device that allows users to have continuous access to their blood sugar levels without having to visually attend to a screen.

More broadly, one could also make a device to expand one's sense of a partner's wellbeing. By connecting sensors that detect a partner's breathing rate, temperature, galvanic skin response, heart rate, and more, a haptic device could expand the user's information flow to allow them to feel their partner's internal signals. Interestingly, this sense need not be limited by proximity: the user can sense how their partner is feeling even from across the country, so long as they have an internet connection.

An important question for any haptic sensory device is whether the user is gaining a new sensory experience or is instead consciously processing the incoming haptic information. In the former case, the subjective experience of a temperature sensing wristband would be similar to the subjective experience of touching a hot stove (without needing to touch or be near the surface), while the latter case would be closer to feeling an alert on the wrist that warns of a hot surface. As mentioned above, previous work has shown evidence for a direct subject experience of a new sense, whether from brain imaging or through subjective questionnaires (Bach-y-Rita et al., 2003; Nagel et al., 2005). Until this investigation is done on each new sensory device, it is unknown whether the device is providing a new sense or rather a signal that can be consciously perceived as touch.

While these devices all expand on one existing sense or another, other devices can go further, representing entirely new senses. These devices form the third group: sensory addition.

## Sensory addition

Due to the brain's remarkable flexibility, there is the possibility of leveraging entirely new data streams directly into perception (Hawkins and Blakeslee, 2007; Eagleman, 2015).

One increasingly common example is the implantation of small neodymium magnets into the fingertips. By this method, “biohackers” can haptically feel magnetic fields. The magnets tug when exposed to electromagnetic fields, and the nearby touch nerves register this. Information normally invisible to humans is now streamed to the brain via the sensory nerves from the fingers. People report that detecting electromagnetic fields (e.g., from a power transformer) is like touching an invisible bubble, one with a shape that can be assessed by moving one's hand around (Dvorsky, 2012). A world is detectable that previously was not: palpable shapes live around microwave ovens, computer fans, speakers, and subway power transformers.

Can haptic devices achieve the same outcome without implanting magnets into the fingertips? One developer created a sensory addition device using a haptic wristband that translates electromagnetic fields into vibrations (details of the project at neosensory.com/developers). Not only is such an approach less invasive, it is also more customizable. Instead of just feeling the presence of an electromagnetic field, this device decomposes the frequency of an alternating current signal and presents the intensity of different parts of the spectrum via different vibrating motors. Thus, an electrician can add this new sense to their perception, knowing the frequency and intensity of electric signals flowing through live wires.

What if you could detect not only the magnetic field around objects but also the one around the planet—as many animal species do? Researchers at Osnabrück University devised a belt called the feelSpace to allow humans to tap into that signal. The belt is ringed with vibratory motors, and the motor pointed to the north buzzes. As you turn your body, you always feel the buzzing in the direction of magnetic north.

At first, it feels like buzzing, but over time it becomes spatial information: a feeling that north is *there* (Kaspar et al., 2014). Over several weeks, the belt changes how people navigate: their orientation improves, they develop new strategies, they gain a higher awareness of the relationship between different places. The environment seems more ordered. Relationships between places can be easily remembered.

As one subject described the experience, “The orientation in the cities was interesting. After coming back, I could retrieve the relative orientation of all places, rooms and buildings, even if I did not pay attention while I was actually there” (Nagel et al., 2005). Instead of thinking about a sequence of cues, they thought about the situation globally. Another user describes how it felt: “It was different from mere tactile stimulation, because the belt mediated a spatial feeling.... I was intuitively aware of the direction of my home or of my office.” In other words, his experience is not of sensory substitution, nor is it sensory expansion (making your sight or hearing better). Instead, it's a sensory addition. It's a new kind of human experience. The user goes on:

During the first 2 weeks, I had to concentrate on it; afterwards, it was intuitive. I could even imagine the arrangement of places and rooms where I sometimes stay. Interestingly, when I take off the belt at night I still feel the vibration: When I turn to the other side, the vibration is moving too—this is a fascinating feeling! (Nagel et al., 2005).

After users take off the feelSense belt, they often report that they continue having a better sense of orientation for some time. In other words, the effect persists even without wearing the device. As with the balance helmet, weak internal signals can get strengthened when an external device confirms them. (Note that one won't have to wear a belt for long: researchers have recently developed a thin electronic skin—essentially a little sticker on the hand—that indicates north; see Bermúdez et al., 2018).

Other projects tackle tasks that require a great deal of cognitive load. For example, a modern aircraft cockpit is packed with visual instruments. With a sensory addition device, a pilot can feel the high-dimensional stream of data instead of having to read all the data visually. The North Atlantic Treaty Organization (NATO) released a report on haptic devices developed to help pilots navigate in low-visibility settings (Van Erp and Self, 2008). Similarly, Fellah and Guiatni (2019) developed a haptic sensory substitution device to give pilots access to the turn rate angle, climb angle, and flight control warning messages via vibrations.

As a related example, researchers at our laboratory are piloting a system to allow doctors to sense the vitals of a patient without having to visually consult a variety of monitors. This device connects a haptic wristband to an array of sensors that measure body temperature, blood oxygen saturation, heart rate, and heart rate variability. Future work will optimize how these data streams are presented to the doctor and with what resolution. Both psychophysical testing and understanding user needs will shape this optimized mapping (for example, how much resolution can a doctor learn to feel in the haptic signal, and what is the smallest change in blood pressure that should be discernible via the device?).

Finally, it is worth asking whether haptic devices are optimal for sending data streams to the brain. After all, one could leverage a higher-resolution sense, such as vision or audition, or perhaps use multiple sensory modalities. This is an open question for the future; however, haptics is advantageous due simply to the fact that vision and hearing are necessary for so many daily tasks. Skin is a high-bandwidth, mostly unused information channel—and therefore its almost-total availability makes it an attractive target for new data streams.

## Conclusion

We have reviewed some of the projects and possibilities of non-invasive, haptic devices for passing new data streams to the brain. The chronic rewiring of the brain gives it tremendous flexibility: it dynamically reconfigures itself to absorb and interact with data. As a result, electrical grids can come to feed visual information via the tongue, vibratory motors can feed hearing via the skin, and cell phones can feed video streams via the ears. Beyond sensory substitution, such devices can be used to endow the brain with new capacities, as we see with sensory expansion (extending the limits of an already-existing sense) and sensory addition (using new data streams to create new senses). Haptic devices have moved rapidly from computer-laden cabled devices to wireless wearables, and this progress, more than any change in the fundamental science, will increase their usage and study.

## Data availability statement

The original contributions presented in the study are included in the article/supplementary material, further inquiries can be directed to the corresponding author.

## Ethics statement

The studies involving human participants were reviewed and approved by Solutions IRB. The patients/participants provided their written informed consent to participate in this study.

## Author contributions

DE wrote the manuscript. MP engineered many of the devices described. Both authors contributed to the article and approved the submitted version.
